# The transcriptome difference between colorectal tumor and normal tissues revealed by single-cell sequencing

**DOI:** 10.7150/jca.32267

**Published:** 2019-10-11

**Authors:** Guo-Liang Zhang, Le-Lin Pan, Tao Huang, Jin-Hai Wang

**Affiliations:** 1 Department of Colorectal Surgery, The First Affiliated Hospital, College of Medicine, Zhejiang University, Hangzhou 310003, Zhejiang, China; 2 Shanghai Institute of Nutrition and Health, Shanghai Institutes for Biological Sciences, Chinese Academy of Sciences, Shanghai 200031, China

**Keywords:** colorectal cancer, single-cell sequencing, transcriptome, support vector machine, minimal redundancy maximal relevance, incremental feature selection

## Abstract

The previous cancer studies were difficult to reproduce since the tumor tissues were analyzed directly. But the tumor tissues were actually a mixture of different cancer cells. The transcriptome of single-cell was much robust than the transcriptome of a mixed tissue. The single-cell transcriptome had much smaller variance. In this study, we analyzed the single-cell transcriptome of 272 colorectal cancer (CRC) epithelial cells and 160 normal epithelial cells and identified 342 discriminative transcripts using advanced machine learning methods. The most discriminative transcripts were LGALS4, PHGR1, C15orf48, HEPACAM2, PERP, FABP1, FCGBP, MT1G, TSPAN1 and CKB. We further clustered the 342 transcripts into two categories. The upregulated transcripts in CRC epithelial cells were significantly enriched in Ribosome, Protein processing in endoplasmic reticulum, Antigen processing and presentation and p53 signaling pathway. The downregulated transcripts in CRC epithelial cells were significantly enriched in Mineral absorption, Aldosterone-regulated sodium reabsorption and Oxidative phosphorylation pathways. The biological analysis of the discriminative transcripts revealed the possible mechanism of colorectal cancer.

## Introduction

Colorectal cancer (CRC) is a major human digestive tract tumor throughout the world and the incidence increases with increasing age 1. According to the latest world health organization (WHO) statistics, colorectal cancer is the third most common malignancy, second only to lung cancer and gastric cancer 2. The occurrence of colorectal cancer is caused by many factors, such as heredity and environment, which is a complicated process involving multiple transcripts and stages.

Pathogenic mechanisms of CRC are clinically important because they are associated with the patient's prognosis and response to treatment 1. The pathogenesis leading to colorectal cancer can be included in following types: chromosomal instability (CIN), microsatellite instability (MSI)/mismatch repair (MMR) and CpG island methylator phenotype (CIMP) 3.

Sufficient evidence has been shown that abnormal signal transduction exists in the initiation and progression of tumor. Cell signal transduction pathways associated with colorectal cancer mainly include Wnt-β-catenin, PI3K/Akt and TGF-β signaling pathway 4-6. On the other hand, transcripts like c-MYC, KRAS, BRAF, PIK3CA, SMAD2 and SMAD4 can also be considered as predictive biomarkers for patient's prognosis 7.

The single-cell transcriptome sequencing is a newly developed technology and measures the sum of all the RNA in a particular cell 8. Through high throughput sequencing, it is possible to obtain almost all transcriptional sequence information of a specific tissue or organ comprehensively and rapidly. This technology has been widely used in the fields of basic research, clinical diagnosis and drug development 9. Moreover, it can also be used for tumor heterogeneity research and the discovery of aberrant proliferative cell types to look for new pathogenesis and mechanisms 10.

Intestinal epithelial cells act as an important barrier to prevent bacterial endotoxin and other toxin into human body. The intestinal epithelium is composed of at least seven different cell types 11, the main function of which is the absorption of nutrients, toxins and drugs. Recent studies suggest that intestinal epithelial cells play an important role in maintaining the intestinal immune homeostasis 12, and the aberrant cell signaling in epithelial junctions has been reported to be associated with the development of colorectal cancer 13.

We analyzed the single-cell transcriptome of 272 CRC epithelial cells and 160 normal epithelial cells. With advanced feature selection methods, we identified 342 discriminative transcripts that showed transcript expression difference between colorectal tumor and normal cells. We found that the upregulated transcripts in CRC epithelial cells were significantly enriched in Ribosome, Protein processing in endoplasmic reticulum, Antigen processing and presentation and p53 signaling pathway while the downregulated transcripts in CRC epithelial cells were significantly enriched in Mineral absorption, Aldosterone-regulated sodium reabsorption and Oxidative phosphorylation pathways. Several identified transcripts, such as LGALS4, FABP1, MT1G, TSPAN1 and CKB, showed great promises as candidates for CRC diagnosis and therapy.

## Materials and Methods

### The single-cell transcriptome of CRC and normal epithelial cells

We downloaded the processed FPKM (Fragments Per Kilobase of transcript per Million mapped reads) single-cell transcriptome of 272 CRC epithelial cells and 160 normal epithelial cells from GEO (Transcript Expression Omnibus) database under accession number of GSE81861 14. Li et al. 14 collected the normal mucosa and CRC tissue and performed single cell sequencing. There were Myeloid, B cell, T cell, Mast, Endo and Epithelial cells in these colorectal tissues. Since most of them were epithelial cells, we focused on epithelial cells. All the data we used have passed the criteria of NODG (number of detected genes) ≥ 1,000, ROER (rate of exonic reads) ≥ 5% and ER (exonic reads) ≥ 0.1 million. More information of data quality control can be found in Li et al. 14

Our goal is to identify the discriminative transcripts using machine learning methods. The 272 CRC epithelial cells were considered as positive samples and 160 normal epithelial cells were considered as negative samples. To filter the noisy transcripts, we only kept the 32,610 transcripts with maximum FPKM (Fragments Per Kilobase Million) across the CRC epithelial cells and normal epithelial cells greater than 5, as features.

### The minimal Redundancy Maximal Relevance method

The mutation information based mRMR (minimal Redundancy Maximal Relevance) method (http://home.penglab.com/proj/mRMR/) 15 was originally developed to analyze image data but then it showed great power in selecting discriminative features in various areas 16-21.

Let us use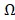 to denote all the 32,610 transcripts, 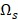 to denote the selected m transcripts, and 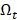 to denote the to-be-selected n transcripts. The relevance 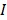 of transcript 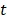 from 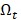
with cell type 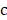 was calculated with mutual information (I) equation 22, 23:


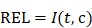
(1)

The redundancy R of the transcript 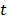 from 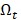
with the selected transcripts in 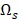
are


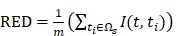
(2)

The goal is to select the transcript 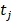 from 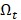
that has maximum relevance with cell type 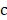 and minimum redundancy with the selected transcripts in
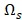



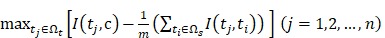
(3)

When 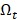 becomes empty, all the transcripts are ranked


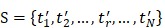
(4)

The rank can represent the discriminating ability of the transcript. Since the mRMR have already reduced the redundancy, the discriminative transcripts will be compact. We focused on the top 500 mRMR transcripts for further analysis.

### Incremental Feature Selection method

To determine how many mRMR transcripts should be selected, Incremental Feature Selection (IFS) method 24-30 was applied. As a wrapped feature selection method, IFS method evaluated the performances of SVM (Support Vector Machine) classifiers constructed based on different transcript combinations. We used the function svm with default parameters in R package e1071 (https://CRAN.R-project.org/package=e1071) to construct the SVM classifier.

Since the transcripts have been ranked using mRMR, it is unnecessary to try all transcript combinations. As a greedy optimization method, each time, one transcript was added into the previous transcript set 31-34 and the classification performance of the updated transcript set was evaluated with leave-one-out cross validation (LOOCV).

The Sensitivity (Sn), Specificity (Sp), Accuracy (ACC) and Mathew's correlation coefficient (MCC) were used to evaluate the prediction performance:


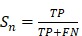
(5)


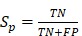
(6)


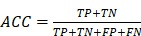
(7)


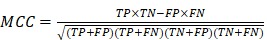
(8)

where TP, TN, FP and FN were the number of true positive, true negative, false positive and false negative samples.

With the performances of all possible IFS combinations, an IFS curve was plotted to visually select the optimized transcript combination. The x-axis was the number of used transcripts and the y-axis was the LOOCV performance. Since the sample size of CRC epithelial cells and normal epithelial cells were quite different, we used the MCC as the major performance evaluator. The peak of IFS curve indicated that the optimal transcript set with highest MCC.

### The up and down regulated transcripts in CRC epithelial cells

The mRMR and IFS methods can identify the transcripts that can classify the CRC epithelial cells and normal epithelial cells, but they can't tell which transcripts were upregulated or downregulated in CRC epithelial cells. To visually investigate the transcript-cell relationship, we applied two-way hierarchical clustering of both CRC/normal epithelial cells and selected transcripts. From the heatmap, we can not only explore whether the CRC and normal epithelial cells were clustered into different groups, but also know which transcripts were upregulated or downregulated in CRC epithelial cells.

## Results

### The transcripts were ranked with mRMR method

The mRMR method ranks the transcripts based on both their relevance with the cell types and their redundancy with other transcripts. Since it considered the redundancy, the selected transcripts will be representative and a small number of top transcripts will be discriminative for cell types. We identified the top 500 most discriminative transcripts using the mRMR method. These 500 transcripts had enough power to discriminate cell types.

### The optimal transcript combinations were identified with IFS method

**Figure 1:**
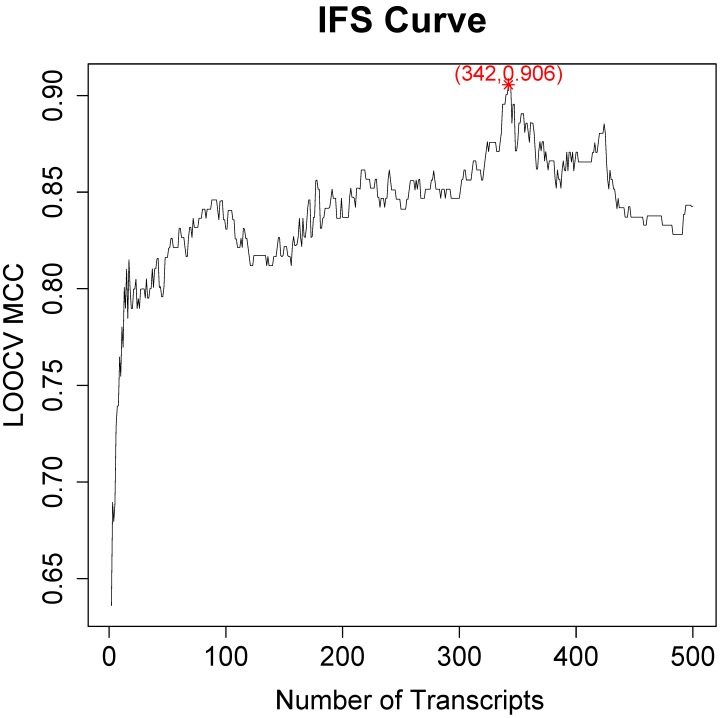
** The IFS curve of how the classifiers based on different number of transcripts performance.** The x-axis was the number of transcripts used to build the classifier and y-axis was the prediction MCC evaluated with LOOCV. The peak of IFS curve was MCC of 0.906 when 342 transcripts were used.

Since the mRMR result was only transcript ranks, it is still difficult to determine how many top transcripts should be chosen. As a greedy optimization method, the IFS method can quickly discovery the optimal transcript combinations with great performance. We tried different combinations of top transcripts and recorded the performance of the SVM classifier constructed based on these transcripts. Then the IFS curve was plotted in **Figure 1** to visually select the transcript combinations. In the IFS curve, the x-axis was the number of transcripts and the y-axis was the LOOCV MCC.

It was found that when the top 342 transcripts were used, the MCC was the highest. The sensitivity, specificity, accuracy and MCC of the 342-transcript classifier were 0.967, 0.938, 0.956 and 0.906, respectively. The 342 selected transcripts were given in **Table S1**.

### The biological analysis of top transcripts

There was local peak in **Figure 1** with MCC around 0.8 when top 20 transcripts were used. Since 342 transcripts were too much to analyze one-by-one, we analyzed the top 20 transcripts which were given in **Table 1**.

The first transcript was LGALS4 which predominantly expressed in small intestine, colon, and rectum, and was under expressed in colorectal cancer. It acts as a tumor suppressor in colorectal carcinoma and suppresses cancer cell growth, migration, and invasion 35. It is a dual function protein: promote cell proliferation and chemokine secretion in galectin-4-expressing colorectal cancer cells, but induce apoptosis in galectin-4-negative colorectal cancer cells 36.

The second transcript was PHGR1 which has been reported to play an essential role in gastrointestinal epithelium and has demonstrated potentials for clinical application in colorectal cancer lymph node metastases detection 37.

The third transcript was C15orf48. It was mainly expressed in esophagus, stomach, small intestine, colon and placenta. The associations between and squamous cell carcinoma has been reported 38.

The fourth transcript was HEPACAM2, a protein of the immunoglobulin superfamily, which plays a role in mitosis. Its expression level was increased in adenomas, the benign stage of tumor glandular tissues, such as the mucosa of small intestine and colon 39. It seems to be involved in cell-cell adhesion and play an important role in tumor metastasis 39.

Another top promising transcript was PERP. It is the component of intercellular desmosome junctions and plays a role in cell-cell adhesion and stratified epithelial integrity. It is involved in p53 Pathway in CRC 40.

FABP1 ranked 10^th^ and encoded the fatty acid binding protein. It is down regulated in colorectal carcinogenesis and associated with poorer prognosis. Lower expression of FABP1 indicated liver metastasis of CRC. FABP1 expression was observed throughout cancer development 41.

The 13^th^ transcript was FCGBP. The FCGBP expression significantly decreased the overall survival of CRC patients and may be a potential therapeutic target for metastatic CRC patients 42.

The 15^th^ transcript MT1G was related to metabolism and response to metal ions. It is silenced through epigenetic mechanisms during colorectal cancer progression, and its loss is associated with poor survival of CRC 43.

TSPAN1, a member of the transmembrane 4 superfamily, ranked 18^th^. The expression level of TSPAN1 is increased in colorectal carcinoma and is an independent prognostic factor for the colorectal adenocarcinoma patients 44. It can be regulated by miR-638 which inhibits TSPAN1 and serve as a tumor suppressor 45.

The 20^th^ transcript in **Table 1** was CKB. Interestingly, CKB is overexpressed in most cancer types, but not in CRC. In CRC, CKB is downregulated. The downregulation of CKB promotes EMT and accelerate colon cancer progression 46.

### The transcripts were up or down regulated in CRC epithelial cells

**Figure 2:**
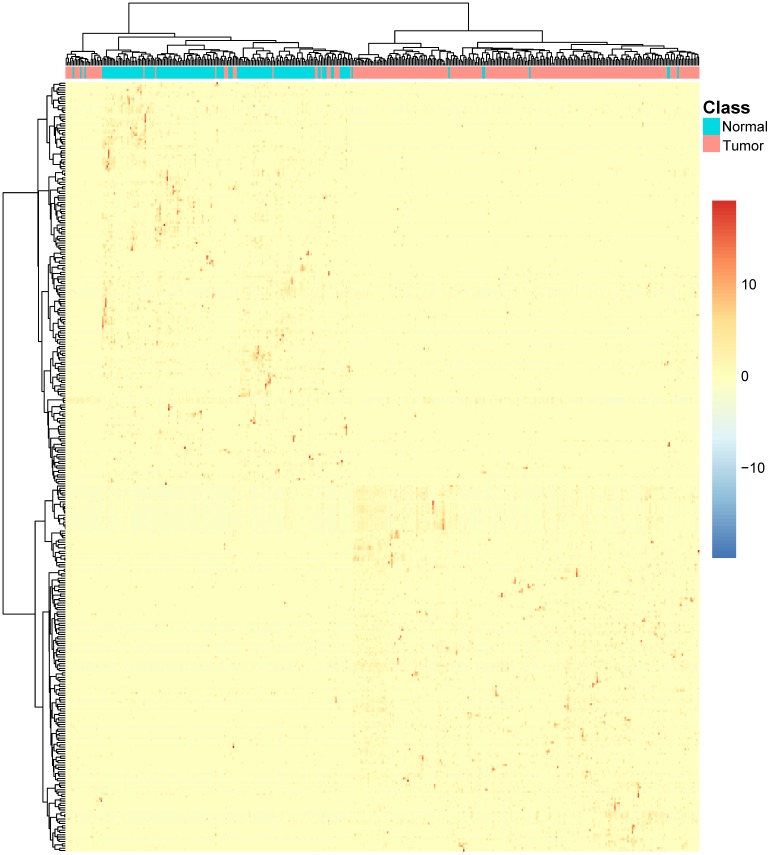
** The heatmap of the 342 transcripts in CRC epithelial cells and normal epithelial cells.** Each row corresponded to the scaled transcript expressed level of a transcript. The warmer colors indicated higher expression level and the colder colors indicated lower expression levels. Each column corresponded to an epithelial cell. The red ones were tumor epithelial cells and the green ones were normal epithelial cells. It can be seen that the CRC epithelial cells and normal epithelial cells were clearly clustered into two groups and correspondingly, the 342 transcripts were also clustered into two groups. The top cluster of transcripts were highly expressed in normal epithelial cells and the bottom cluster of transcripts were highly expressed in CRC epithelial cells.

To intuitively explore the transcript-cell relationship, we plotted two-way hierarchical clustering of both CRC/normal epithelial cells and 342 transcripts in **Figure 2**. It can be seen that the CRC epithelial cells and normal epithelial cells were clearly clustered into two groups and correspondingly, the 342 transcripts were also clustered into two groups. The top cluster of transcripts were highly expressed in normal epithelial cells and the bottom cluster of transcripts were highly expressed in CRC epithelial cells.

We enriched the up regulated transcripts and down regulated transcripts onto KEGG pathway and GO terms using hypergeometric test 47-53.

The significantly enriched KEGG pathways of the up regulated transcripts in CRC epithelial cells was given in **Table 2**. It can be seen that Ribosome, Protein processing in endoplasmic reticulum, Antigen processing and presentation, p53 signaling pathway were enriched.

The significantly enriched KEGG pathways of the down regulated transcripts in CRC epithelial cells was given in **Table 3**. It can be seen that in CRC epithelial cells, the activity of Mineral absorption, Aldosterone-regulated sodium reabsorption and Oxidative phosphorylation were decreased. Han et al. have also reported that the differentially expressed genes (DEGs) of Colorectal cancer were enriched in mineral absorption 54.

The significantly enriched GO biological process (BP), molecular function (MF) and cellular component (CC) terms of the up regulated transcripts in CRC epithelial cells was given in **Table S2**. The significantly enriched GO biological process (BP), molecular function (MF) and cellular component (CC) terms of the down regulated transcripts in CRC epithelial cells was given in **Table S3**.

### The network of the key transcripts in CRC epithelial cells

**Figure 3:**
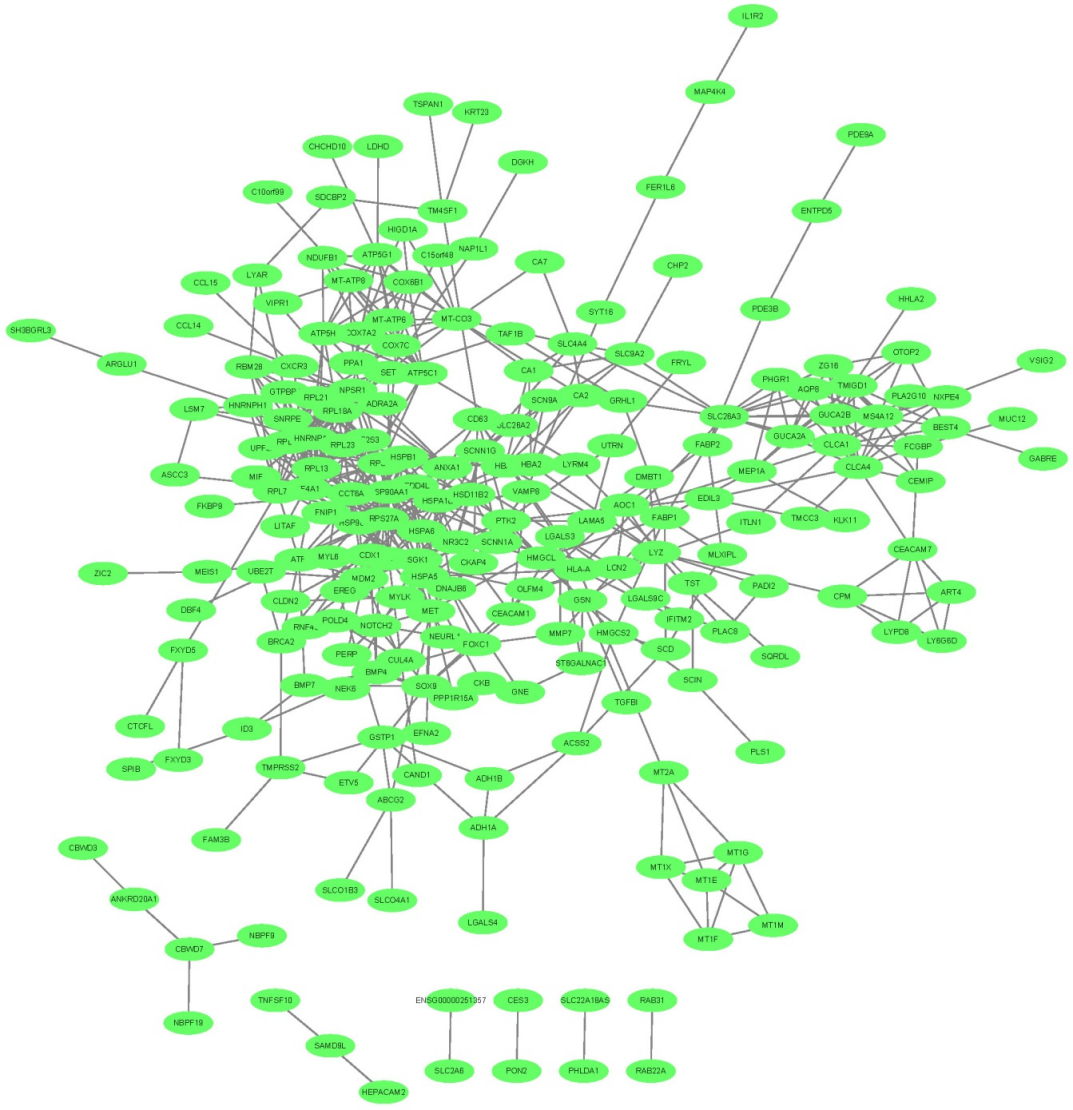
** The network of the key transcripts in CRC epithelial cells**. The 342 key transcripts in CRC epithelial cells were mapped onto STRING network. The 280 mapped genes had 578 interactions which were much more than expected with PPI (Protein-Protein Interaction) enrichment p-value smaller than 1.0e-16. They were closely connected.

We mapped the 342 key transcripts in CRC epithelial cells onto STRING network 55 and constructed their interaction network with confidence score greater than 0.4. The network was shown in **Figure 3**. 280 genes can be mapped and they had 578 interactions which were much more than expected 349 edges with PPI (Protein-Protein Interaction) enrichment p-value smaller than 1.0e-16. They were biologically connected as a group.

### Compare the key transcripts with other CRC signature genes

We compared the 342 key transcripts with other CRC signature genes. Chu et al. did a meta-analysis of the differentially expressed genes between colorectal tumors and normal mucosa in 16 datasets and identified a 55-gene CRC signature 56. **Table S4** listed the 55 CRC signature genes from Chu et al. 56. We did hypergeometric test of the overlap between the 55 CRC signature genes and our 342 genes. There were 22 overlapped genes: ABCG2, AQP8, CA1, CA7, CDH3, CHP2, CLCA1, CLCA4, CPM, FCGBP, GUCA2A, GUCA2B, KIAA1199, KLK11, MMP7, MS4A12, MT1M, NR3C2, SLC26A3, SLC4A4, SPIB, ZG16. The p-value was 2.2e-30 and the odds ratio was 75.5. Their overlap was very significant.

## Discussion

Overall, in CRC epithelial cells, the biological processes of SRP-dependent co-translational protein targeting to membrane, co-translational protein targeting to membrane, protein targeting to ER, establishment of protein localization to endoplasmic reticulum, negative regulation of cell cycle arrest, response to oxidative stress, negative regulation of programmed cell death, regulation of cellular response to stress, negative regulation of cell death, regulation of cell cycle process, regulation of cell cycle, cellular response to stress, ribosome biogenesis and cell death were enriched for the up regulated transcripts. Many of them were typical cancer related pathways. For the down regulated transcripts in CRC epithelial cells, they were enriched onto cellular response to zinc ion, response to zinc ion, cellular response to cadmium ion and digestion biological processes. These were epithelial cell specific functions in normal tissues but disrupted in tumor tissue.

Tumor heterogeneity is a key issue for cancer diagnosis and treatment. The traditional analysis of tumor tissues from cancer patients are usually difficult to reproduce since the tumor tissue is a mixture of different cells. The single cell sequencing enables the gene expression profiles on cell level. In this study, we analyzed the single-cell transcriptome of CRC epithelial cells and normal epithelial cells and identified the differentially expressed transcripts using advanced machine learning methods. It was found that the upregulated transcripts in CRC epithelial cells were significantly enriched in Ribosome, Protein processing in endoplasmic reticulum, Antigen processing and presentation and p53 signaling pathway while the downregulated transcripts in CRC epithelial cells were significantly enriched in Mineral absorption, Aldosterone-regulated sodium reabsorption and Oxidative phosphorylation pathways. The biological analysis of selected transcripts revealed the possible mechanism of colorectal cancer.

## Supplementary Material

Supplementary tables.

## Figures and Tables

**Table 1:** The top 20 mRMR transcripts

Order	Chromosome	Start Position	End Position	Transcript Name	Transcript ID	Score
1	chr19	39292310	39304004	LGALS4	ENSG00000171747.4	0.138
2	chr15	40643233	40648635	PHGR1	ENSG00000233041.4	0.085
3	chr15	45722726	45878488	C15orf48	ENSG00000166920.6	0.067
4	chr7	92817898	92855837	HEPACAM2	ENSG00000188175.5	0.065
5	chr16	56659386	56661024	MT1E	ENSG00000169715.10	0.066
6	chr6	138409641	138428648	PERP	ENSG00000112378.11	0.061
7	chr10	85933493	85945050	C10orf99	ENSG00000188373.4	0.063
8	chr1	45249256	45253377	BEST4	ENSG00000142959.4	0.059
9	chr13	27825445	27830828	RPL21	ENSG00000122026.6	0.054
10	chr2	88422509	88427635	FABP1	ENSG00000163586.5	0.055
11	chr12	39040623	39303394	CPNE8	ENSG00000139117.9	0.054
12	chr12	56544579	56584068	MYL6	ENSG00000092841.14	0.053
13	chr19	40353962	40440533	FCGBP	ENSG00000090920.9	0.052
14	chr20	1290618	1373806	SDCBP2	ENSG00000125775.10	0.053
15	chr16	56700642	56701977	MT1G	ENSG00000125144.9	0.053
16	chr6	31795511	31798031	HSPA1B	ENSG00000204388.5	0.052
17	chr5	179041178	179061785	HNRNPH1	ENSG00000169045.13	0.051
18	chr1	46505811	46651630	TSPAN1	ENSG00000117472.5	0.051
19	chr16	56662970	56667898	MT1M	ENSG00000205364.3	0.051
20	chr14	103985995	103989448	CKB	ENSG00000166165.8	0.051

**Table 2:** The significantly enriched KEGG pathways of the up regulated transcripts in CRC epithelial cells

KEGG pathway	FDR*	P value	Transcripts
hsa03010 Ribosome	0.00136	4.32E-06	RPS4Y1, RPS18, RPS27A, RPL7, RPL13, RPL18A, RPL21, RPL23, RPL38
hsa04141 Protein processing in endoplasmic reticulum	0.0456	0.000422	HSPA5, HSPA1B, HSPA6, HSP90AA1, HSP90AB1, HSPH1, PPP1R15A
hsa04612 Antigen processing and presentation	0.0456	0.000436	HSPA1B, HSPA6, HSP90AA1, HSP90AB1, HSPA5
hsa05215 Prostate cancer	0.0981	0.00125	ETV5, MDM2, HSP90AA1, HSP90AB1, GSTP1
hsa04115 p53 signaling pathway	0.153	0.00244	ATR, MDM2, PERP, SESN3

*: FDR<0.2

**Table 3:** The significantly enriched KEGG pathways of the down regulated transcripts in CRC epithelial cells

KEGG pathway	FDR*	P value	Transcripts
hsa04978 Mineral absorption	0.000195	6.21E-07	SLC26A3, MT1E, MT1F, MT1G, MT1M, MT1X, MT2A
hsa04960 Aldosterone-regulated sodium reabsorption	0.000236	1.50E-06	HSD11B2, NR3C2, SCNN1A, SCNN1G, SGK1, NEDD4L
hsa00190 Oxidative phosphorylation	0.0295	0.000347	NDUFB1, COX6B1, COX7A2, COX7C, ATP5C1, ATP5G1, ATP5H
hsa04972 Pancreatic secretion	0.029	0.000376	PLA2G10, CLCA1, CLCA4, SLC26A3, SLC4A4, CA2
hsa05012 Parkinson's disease	0.0311	0.000516	NDUFB1, COX6B1, COX7A2, COX7C, ATP5C1, ATP5G1, ATP5H
hsa00910 Nitrogen metabolism	0.0311	0.000595	CA1, CA7, CA2

*: FDR<0.05
