# Supplementary material for: The transcriptome difference between colorectal tumor and normal tissues revealed by single-cell sequencing
Source: J Cancer. 2019 Oct 11;10(23):5883–90. doi: 10.7150/jca.32267 (PMC6843882; doi:10.7150/jca.32267)
Supplement: Supplementary file 1 — Supplementary tables. [file jcav10p5883s1.pdf]

Table S1 - The top 342 transcripts

Order	Chromosome Position	Transcript Name	Transcript ID	Score
1	chr19:39292310-39304004	LGALS4	ENSG00000171747.4	0.138
2	chr15:40643233-40648635	PHGR1	ENSG00000233041.4	0.085
3	chr15:45722726-45878488	C15orf48	ENSG00000166920.6	0.067
4	chr7:92817898-92855837	HEPACAM2	ENSG00000188175.5	0.065
5	chr16:56659386-56661024	MT1E	ENSG00000169715.10	0.066
6	chr6:138409641-138428648	PERP	ENSG00000112378.11	0.061
7	chr10:85933493-85945050	C10orf99	ENSG00000188373.4	0.063
8	chr1:45249256-45253377	BEST4	ENSG00000142959.4	0.059
9	chr13:27825445-27830828	RPL21	ENSG00000122026.6	0.054
10	chr2:88422509-88427635	FABP1	ENSG00000163586.5	0.055
11	chr12:39040623-39303394	CPNE8	ENSG00000139117.9	0.054
12	chr12:56544579-56584068	MYL6	ENSG00000092841.14	0.053
13	chr19:40353962-40440533	FCGBP	ENSG00000090920.9	0.052
14	chr20:1290618-1373806	SDCBP2	ENSG00000125775.10	0.053
15	chr16:56700642-56701977	MT1G	ENSG00000125144.9	0.053
16	chr6:31795511-31798031	HSPA1B	ENSG00000204388.5	0.052
17	chr5:179041178-179061785	HNRNPH1	ENSG00000169045.13	0.051
18	chr1:46505811-46651630	TSPAN1	ENSG00000117472.5	0.051
19	chr16:56662970-56667898	MT1M	ENSG00000205364.3	0.051
20	chr14:103985995-103989448	CKB	ENSG00000166165.8	0.051
21	chr17:46952221-47006418	ATP5G1	ENSG00000159199.9	0.05
22	chr2:231729353-231743963	ITM2C	ENSG00000135916.11	0.049
23	chr5:149340299-149432386	SLC26A2	ENSG00000155850.7	0.049
24	chr9:131445702-131458679	SET	ENSG00000119335.12	0.048
25	chr16:29789560-29793096	ZG16	ENSG00000174992.6	0.046
26	chr1:42628361-42630389	GUCA2A	ENSG00000197273.3	0.046
27	chr14:102547074-102771537	HSP90AA1	ENSG00000080824.14	0.045
28	chr17:37004117-37010096	RPL23	ENSG00000125691.8	0.045
29	chr2:169921298-169952677	DHRS9	ENSG00000073737.12	0.044
30	chr13:111530886-111567416	ANKRD10	ENSG00000088448.10	0.043
31	chr1:17393255-17445948	PADI2	ENSG00000117115.8	0.044
32	chr11:307630-315272	IFITM2	ENSG00000185201.12	0.043
33	chr3:42734154-42846023	HIGD1A	ENSG00000181061.9	0.043
34	chr16:23765947-23770272	CHP2	ENSG00000166869.2	0.042
35	chr19:35645632-35660786	FXYD5	ENSG00000089327.10	0.042
36	chr14:21269386-21271437	RNASE1	ENSG00000129538.9	0.042
37	chr1:248902715-248903150	LYPD8	ENSG00000266949.1	0.042
38	chr11:60260250-60274903	MS4A12	ENSG00000071203.5	0.042
39	chrX:24072832-24096088	EIF2S3	ENSG00000130741.6	0.042
40	chr11:67118247-67141648	POLD4	ENSG00000175482.4	0.04
41	chr1:120290618-120311528	HMGCS2	ENSG00000134240.7	0.04
42	chr10:102106880-102124591	SCD	ENSG00000099194.5	0.039
43	chr14:106109388-106115394	IGHG2	ENSG00000211893.3	0.039
44	chr1:212208918-212280742	RPL21P28	ENSG00000220749.3	0.039
45	chr15:51633825-51700210	GLDN	ENSG00000186417.9	0.038
46	chr16:56642110-56643409	MT2A	ENSG00000125148.6	0.038
47	chr13:31710761-31736525	HSPH1	ENSG00000120694.15	0.038
48	chr9:72999502-73029540	KLF9	ENSG00000119138.3	0.038
49	chr7:128095893-128146656	HILPDA	ENSG00000135245.9	0.037
50	chr11:124617367-124635832	VSIG2	ENSG00000019102.7	0.037
51	chr4:148999912-149365850	NR3C2	ENSG00000151623.10	0.036
52	chr13:107194020-107220512	ARGLU1	ENSG00000134884.9	0.036
53	chr17:70017991-70216859	SOX9	ENSG00000125398.5	0.036

54	chr16:56691605-56694610	MT1F	ENSG00000198417.5	0.037
55	chr9:123940414-124095121	GSN	ENSG00000148180.12	0.036
56	chr8:86239836-86393722	CA2	ENSG00000104267.5	0.037
57	chr20:44095909-44110172	WFDC2	ENSG00000101443.13	0.036
58	chr14:55590827-55612126	LGALS3	ENSG00000131981.11	0.036
59	chr7:107405911-107443670	SLC26A3	ENSG00000091138.8	0.036
60	chr12:69201955-69365350	CPM	ENSG00000135678.7	0.036
61	chr4:77870855-77961537	SEPT11	ENSG00000138758.7	0.036
62	chr9:69204537-69269662	CBWD6	ENSG00000204790.8	0.036
63	chr8:86239836-86393722	CA1	ENSG00000133742.9	0.036
64	chr7:155266900-155326557	AC008060.5	ENSG00000227365.1	0.036
65	chr7:150521714-150558592	AOC1	ENSG00000002726.15	0.035
66	chr19:51515994-51523431	CTC-518B2.12	ENSG00000268739.1	0.035
67	chr19:42177234-42210895	CEACAM7	ENSG00000007306.10	0.036
68	chr12:76438669-76478813	NAP1L1	ENSG00000187109.9	0.035
69	chr17:34310326-34338658	CCL14	ENSG00000213494.5	0.035
70	chr21:44073745-44195619	PDE9A	ENSG00000160191.13	0.035
71	chr1:207925401-207968858	CD46	ENSG00000117335.14	0.035
72	chr7:19958603-20257027	RPL21P75	ENSG00000213860.3	0.035
73	chr12:20963635-21392180	SLCO1B3	ENSG00000111700.8	0.035
74	chr8:145202918-145316843	MROH1	ENSG00000179832.13	0.035
75	chr4:72053002-72437804	SLC4A4	ENSG00000080493.9	0.035
76	chr12:69742120-69748014	RP11-1143G9.4	ENSG00000257764.2	0.035
77	chr17:39077681-39132178	KRT23	ENSG00000108244.12	0.035
78	chr6:134490383-134639250	SGK1	ENSG00000118515.7	0.035
79	chr1:87012760-87158886	CLCA4	ENSG00000016602.8	0.035
80	chr7:116312443-116438440	MET	ENSG00000105976.10	0.034
81	chr22:24198613-24241117	AP000350.10	ENSG00000251357.4	0.035
82	chr17:34310326-34338658	CCL15-CCL14	ENSG00000161574.11	0.034
83	chr14:106202679-106209408	IGHG1	ENSG00000211896.2	0.034
84	chr6:1312674-1314992	FOXQ1	ENSG00000164379.4	0.034
85	chr2:166813908-167232503	SCN9A	ENSG00000169432.10	0.034
86	chr19:35606731-35615228	FXYD3	ENSG00000089356.12	0.034
87	chr9:130911349-130915734	LCN2	ENSG00000148346.7	0.034
88	chr5:135364583-135399507	TGFBI	ENSG00000120708.12	0.034
89	chr7:100612903-100662230	MUC12	ENSG00000205277.5	0.034
90	chr1:40798747-40799230	RP1-228H13.1	ENSG00000227311.2	0.034
91	chr15:44019115-44095241	SERF2	ENSG00000140264.15	0.034
92	chr16:23194035-23228204	SCNN1G	ENSG00000166828.2	0.034
93	chr16:66836777-66907159	CA7	ENSG00000168748.9	0.034
94	chr1:26605666-26647014	SH3BGRL3	ENSG00000142669.9	0.033
95	chr12:76419226-76427712	PHLDA1	ENSG00000139289.9	0.033
96	chr16:222845-223709	HBA2	ENSG00000188536.8	0.033
97	chr6:29909036-29913661	HLA-A	ENSG00000206503.7	0.033
98	chr17:72920369-72930007	OTOP2	ENSG00000183034.8	0.033
99	chr1:23884408-23886285	ID3	ENSG00000117318.8	0.033
100	chr13:115047058-115071283	UPF3A	ENSG00000169062.10	0.033
101	chr3:42530790-42579059	VIPR1	ENSG00000114812.8	0.033
102	chr11:27910384-27912580	RP11-1033A18.1	ENSG00000224411.2	0.033
103	chr20:61272070-61317137	SLCO4A1	ENSG00000101187.11	0.033
104	chr2:102608305-102645006	IL1R2	ENSG00000115590.9	0.032
105	chr8:74202505-74268696	RPL7	ENSG00000147604.9	0.032
106	chr22:37406899-37425863	TST	ENSG00000128311.9	0.032
107	chr11:2016405-2022700	H19	ENSG00000130600.11	0.032

108	chr7:56119322-56131682	CCT6A	ENSG00000146731.6	0.032
109	chr4:120238404-120243545	FABP2	ENSG00000145384.3	0.032
110	chr1:173832385-173872687	GAS5	ENSG00000234741.3	0.032
111	chr3:172223297-172241297	TNFSF10	ENSG00000121858.6	0.031
112	chr1:22138757-22263790	RP11-26H16.1	ENSG00000232037.2	0.031
113	chr12:94960899-95044338	TMCC3	ENSG00000057704.6	0.031
114	chr16:68670091-68756519	CDH3	ENSG00000062038.9	0.031
115	chr12:69742120-69748014	LYZ	ENSG00000090382.2	0.031
116	chr6:75947390-75960039	COX7A2	ENSG00000112695.7	0.031
117	chr17:28643350-28661077	TMIGD1	ENSG00000182271.8	0.031
118	chr2:110841446-110874143	MALL	ENSG00000144063.3	0.031
119	chr1:203830730-203839678	SNRPE	ENSG00000182004.8	0.031
120	chr1:120454175-120612240	NOTCH2	ENSG00000134250.13	0.03
121	chr17:46799083-46799884	PRAC1	ENSG00000159182.3	0.031
122	chrX:102930423-102946700	MORF4L2	ENSG00000123562.12	0.03
123	chr19:2321515-2355099	LSM7	ENSG00000130332.10	0.03
124	chr2:190744334-191236391	C2orf88	ENSG00000187699.6	0.03
125	chr6:44214823-44221620	HSP90AB1	ENSG00000096384.15	0.03
126	chr16:226678-227521	HBA1	ENSG00000206172.4	0.03
127	chr19:50887460-50934570	SPIB	ENSG00000269404.2	0.03
128	chr17:73584138-73704142	SMIM5	ENSG00000204323.5	0.03
129	chr10:71962585-71993667	PPA1	ENSG00000180817.7	0.03
130	chr9:43064965-43133544	ANKRD20A3	ENSG00000132498.7	0.03
131	chr17:73028669-73061984	ATP5H	ENSG00000167863.7	0.03
132	chr4:84011200-84058228	PLAC8	ENSG00000145287.6	0.03
133	chr10:124320180-124403252	DMBT1	ENSG00000187908.11	0.03
134	chr17:72199720-72209481	RPL38	ENSG00000172809.8	0.03
135	chr13:82264045-82265207	PTMAP5	ENSG00000214182.5	0.029
136	chrY:2709526-2800041	RPS4Y1	ENSG00000129824.11	0.029
137	chr1:80916767-80917689	HNRNPA1P64	ENSG00000213559.4	0.029
138	chr16:67464554-67471456	HSD11B2	ENSG00000176387.6	0.029
139	chr12:106631654-106740793	CKAP4	ENSG00000136026.9	0.029
140	chr6:31654725-31685695	LY6G6D	ENSG00000244355.3	0.029
141	chr1:202300784-202311108	UBE2T	ENSG00000077152.5	0.029
142	chr15:81071683-81282219	KIAA1199	ENSG00000103888.11	0.029
143	chr2:66653866-66801001	MEIS1	ENSG00000143995.15	0.029
144	chr20:49348080-49373332	PARD6B	ENSG00000124171.4	0.029
145	chr17:56378591-56494956	RNF43	ENSG00000108375.8	0.029
146	chr13:45007654-45151283	TSC22D1	ENSG00000102804.10	0.029
147	chr18:55711598-56068772	NEDD4L	ENSG00000049759.12	0.029
148	chr14:74318546-74551196	ENTPD5	ENSG00000187097.8	0.029
149	chr7:157128074-157210133	DNAJB6	ENSG00000105993.10	0.029
150	chr3:123304388-123603178	MYLK	ENSG00000065534.14	0.029
151	chr12:104164230-104234975	NT5DC3	ENSG00000111696.7	0.029
152	chr1:26605666-26647014	UBXN11	ENSG00000158062.16	0.029
153	chr9:36190852-36304921	GNE	ENSG00000159921.10	0.029
154	chr1:148003641-148025863	NBPF14	ENSG00000122497.13	0.029
155	chr13:32889610-32973805	BRCA2	ENSG00000139618.10	0.029
156	chr16:67464554-67471456	AC009061.1	ENSG00000203401.4	0.029
157	chr20:33432522-33515769	ACSS2	ENSG00000131069.15	0.029
158	chr6:1610680-1614127	FOXC1	ENSG00000054598.5	0.029
159	chrM:8365-9990	MT-ATP8	ENSG00000228253.1	0.029
160	chr19:35615416-35645204	LGI4	ENSG00000153902.9	0.029
161	chr13:113831890-113919399	CUL4A	ENSG00000139842.10	0.029

162	chr11:114441312-114466484	NXPE4	ENSG00000137634.5	0.029
163	chr2:102313311-102511150	MAP4K4	ENSG00000071054.11	0.028
164	chr9:70856396-70914929	CBWD3	ENSG00000196873.11	0.028
165	chr19:51515994-51523431	KLK10	ENSG00000129451.7	0.028
166	chr20:55743803-55841685	BMP7	ENSG00000101144.8	0.028
167	chr16:14766404-14788526	PLA2G10	ENSG00000069764.5	0.028
168	chr1:86934050-86965972	CLCA1	ENSG00000016490.11	0.028
169	chr19:51525471-51531295	KLK11	ENSG00000167757.9	0.028
170	chr6:33217310-33244287	RPS18	ENSG00000231500.2	0.028
171	chr13:50601268-51423190	DLEU2	ENSG00000231607.4	0.028
172	chr10:18041217-18089855	TMEM236	ENSG00000184040.7	0.028
173	chr16:75145757-75150669	LDHD	ENSG00000166816.9	0.028
174	chr20:52553315-52687304	BCAS1	ENSG00000064787.8	0.028
175	chr1:28832454-28865812	SNHG3	ENSG00000242125.2	0.028
176	chr4:15937191-15940363	FGFBP1	ENSG00000137440.3	0.028
177	chr9:127997131-128003609	HSPA5	ENSG00000044574.7	0.028
178	chr5:83236372-83786583	EDIL3	ENSG00000164176.8	0.028
179	chr1:42619091-42621495	GUCA2B	ENSG00000044012.3	0.028
180	chr2:85788684-85809154	VAMP8	ENSG00000118640.6	0.028
181	chr4:48499377-48782339	FRYL	ENSG00000075539.9	0.027
182	chr12:14956505-15059520	ART4	ENSG00000111339.6	0.027
183	chr1:200374067-200379184	ZNF281	ENSG00000162702.7	0.027
184	chr14:62453802-62568431	SYT16	ENSG00000139973.11	0.027
185	chr6:144606836-145174170	UTRN	ENSG00000152818.14	0.027
186	chr3:108015375-108097132	HHLA2	ENSG00000114455.9	0.027
187	chr13:53602893-53626192	OLFM4	ENSG00000102837.6	0.027
188	chr7:12609750-12693228	SCIN	ENSG00000006747.10	0.027
189	chr8:124864226-125183763	FER1L6	ENSG00000214814.2	0.027
190	chr16:9250218-9250761	RPL21P119	ENSG00000220793.4	0.027
191	chr14:54416453-54425479	BMP4	ENSG00000125378.11	0.027
192	chr9:67926760-67987998	ANKRD20A1	ENSG00000196774.3	0.027
193	chr6:122793075-123047518	PKIB	ENSG00000135549.10	0.027
194	chr22:24105207-24126503	CHCHD10	ENSG00000250479.4	0.027
195	chr1:148250248-148347506	NBPF20	ENSG00000203832.6	0.027
196	chr21:42836477-42903043	TMPRSS2	ENSG00000184012.7	0.027
197	chr16:89627064-89630950	RPL13	ENSG00000167526.9	0.027
198	chr17:34310326-34338658	CCL15	ENSG00000267596.1	0.027
199	chr17:34083267-34122711	MMP28	ENSG00000129270.11	0.027
200	chr4:100226120-100242558	ADH1B	ENSG00000196616.8	0.027
201	chr13:100634025-100639018	ZIC2	ENSG00000043355.6	0.027
202	chr14:92582465-92588261	NDUFB1	ENSG00000183648.5	0.027
203	chr6:31654725-31685695	MEGT1	ENSG00000250641.1	0.027
204	chr6:46761126-46807515	MEP1A	ENSG00000112818.5	0.027
205	chr1:160846328-160854960	ITLN1	ENSG00000179914.4	0.027
206	chr9:75766672-75785309	ANXA1	ENSG00000135046.9	0.027
207	chr5:76506273-76916436	RPL7P23p	ENSG00000244363.2	0.027
208	chr7:100547186-100611410	RP11-395B7.2	ENSG00000225946.1	0.027
209	chr16:4827669-4846533	SMIM22	ENSG00000267795.1	0.027
210	chr16:56716335-56721964	MT1X	ENSG00000187193.8	0.027
211	chr3:185764096-186080026	ETV5	ENSG00000244405.3	0.026
212	chr5:142657495-142815077	RP11-278J6.1	ENSG00000231579.3	0.026
213	chr7:130621222-130624420	RP11-138A9.2	ENSG00000273319.1	0.026
214	chr19:1285889-1378430	EFNA2	ENSG00000099617.2	0.026
215	chr15:45879320-45983492	SQRDL	ENSG00000137767.9	0.026

216	chr7:128095893-128146656	RP11-155G14.6	ENSG00000240758.2	0.026
217	chr3:142168076-142297668	ATR	ENSG00000175054.10	0.026
218	chr20:34633543-34638882	LINC00657	ENSG00000260032.1	0.026
219	chr10:112629500-112679032	BBIP1	ENSG00000214413.3	0.026
220	chr10:122114176-122114718	RP11-572P18.1	ENSG00000220842.5	0.026
221	chr14:106130969-106136180	IGHGP	ENSG00000253755.1	0.026
222	chr12:6456008-6500733	SCNN1A	ENSG00000111319.8	0.026
223	chr16:25227051-25240261	AQP8	ENSG00000103375.6	0.026
224	chr19:36139124-36149763	COX6B1	ENSG00000126267.4	0.026
225	chr7:34386123-34917944	NPSR1	ENSG00000187258.9	0.026
226	chr2:55401926-55462989	RPS27A	ENSG00000143947.8	0.026
227	chr19:31765850-31908700	TSHZ3	ENSG00000121297.6	0.026
228	chr13:27998680-28024739	GTF3A	ENSG00000122034.8	0.026
229	chr11:61976139-62028021	SCGB2A1	ENSG00000124939.4	0.026
230	chr2:9983482-10074545	TAF1B	ENSG00000115750.12	0.026
231	chr11:127810807-127811549	RP11-676M6.1	ENSG00000254612.2	0.025
232	chr17:18380050-18398259	LGALS9C	ENSG00000171916.12	0.025
233	chr4:128702975-128765195	RP11-50D9.1	ENSG00000244021.3	0.025
234	chr4:39500374-39640710	SMIM14	ENSG00000163683.7	0.025
235	chr5:130759613-131132710	FNIP1	ENSG00000217128.7	0.025
236	chr7:92759367-92777682	SAMD9L	ENSG00000177409.7	0.025
237	chr21:42676138-42729358	FAM3B	ENSG00000183844.12	0.025
238	chr10:102133371-102143125	LINC00263	ENSG00000235823.1	0.025
239	chr12:104382761-104457961	RP11-490H24.5	ENSG00000216285.4	0.025
240	chr17:16592850-16719854	UPF3AP1	ENSG00000226478.2	0.025
241	chr16:14821138-14827204	RP11-719K4.3	ENSG00000254609.1	0.025
242	chr1:24128374-24165110	HMGCL	ENSG00000117305.10	0.025
243	chr11:2909009-2946476	SLC22A18AS	ENSG00000254827.1	0.025
244	chr12:56119106-56124467	CD63	ENSG00000135404.7	0.025
245	chr7:32535037-33102409	FKBP9	ENSG00000122642.6	0.025
246	chr7:65216128-65228341	CCT6P1	ENSG00000228409.1	0.025
247	chr10:63661058-63856703	ARID5B	ENSG00000150347.10	0.025
248	chr4:75230859-75254468	EREG	ENSG00000124882.3	0.025
249	chr16:11641852-11730237	LITAF	ENSG00000189067.8	0.025
250	chr17:74620842-74639920	ST6GALNAC1	ENSG00000070526.10	0.025
251	chr6:5102826-5261172	LYRM4	ENSG00000214113.6	0.025
252	chr4:89011415-89152474	ABCG2	ENSG00000118777.6	0.025
253	chr17:1614804-1641893	MIR22HG	ENSG00000186594.8	0.025
254	chrM:8365-9990	MT-ATP6	ENSG00000198899.2	0.025
255	chr19:22235253-22274282	ZNF257	ENSG00000197134.7	0.024
256	chr19:17970684-17974962	RPL18A	ENSG00000105640.8	0.024
257	chr12:69201955-69365350	MDM2	ENSG00000135679.17	0.024
258	chr18:61143993-61172318	RP11-635N19.3	ENSG00000269989.1	0.024
259	chrX:99929487-99987110	SYTL4	ENSG00000102362.11	0.024
260	chr7:155755325-155759037	AC021218.2	ENSG00000204876.4	0.024
261	chr10:105239359-105615301	NEURL	ENSG00000107954.6	0.024
262	chr2:89246818-89247475	IGKV1-5	ENSG00000243466.1	0.024
263	chr22:24198613-24241117	AP000350.4	ENSG00000218537.1	0.024
264	chr1:55446464-55457966	TMEM61	ENSG00000143001.4	0.024
265	chr5:21616370-22853731	HSPD1P1	ENSG00000213430.5	0.024
266	chr10:105239359-105615301	RP11-416N2.4	ENSG00000273108.1	0.024
267	chr16:56651387-56652730	MT1L	ENSG00000260549.1	0.024
268	chr4:100010007-100222513	ADH1A	ENSG00000187758.3	0.024
269	chr1:168338291-168338839	RP5-1059H15.1	ENSG00000227722.1	0.024

270	chr1:144811743-144830413	NBPF9	ENSG00000168614.13	0.024
271	chr1:200890121-200935658	MROH3P	ENSG00000233217.1	0.024
272	chr10:7830091-7849778	ATP5C1	ENSG00000165629.15	0.024
273	chr18:9708161-9862548	RAB31	ENSG00000168461.8	0.024
274	chr2:10085340-10142411	GRHL1	ENSG00000134317.13	0.024
275	chr20:60877148-60942368	LAMA5	ENSG00000130702.9	0.024
276	chrX:129757349-130037208	ENOX2	ENSG00000165675.12	0.024
277	chr12:67663060-67713731	CAND1	ENSG00000111530.8	0.024
278	chr19:42901279-43156507	CEACAM1	ENSG00000079385.17	0.024
279	chr9:127019884-127115586	NEK6	ENSG00000119408.12	0.023
280	chr9:136336216-136344259	SLC2A6	ENSG00000160326.9	0.023
281	chrX:106045909-106243474	CLDN2	ENSG00000165376.6	0.023
282	chr9:21802541-22121096	CDKN2B-AS1	ENSG00000240498.2	0.023
283	chr21:15608526-15735075	ABCC13	ENSG00000243064.4	0.023
284	chr4:4269427-4291896	LYAR	ENSG00000145220.9	0.023
285	chr10:1034337-1095110	GTPBP4	ENSG00000107937.14	0.023
286	chr12:54624723-54745633	HNRNPA1	ENSG00000135486.13	0.023
287	chrX:70835765-70838367	CXCR3	ENSG00000186810.7	0.023
288	chr19:49375648-49379314	PPP1R15A	ENSG00000087074.7	0.023
289	chr8:141667998-142012315	PTK2	ENSG00000169398.15	0.023
290	chr17:7465191-7536700	SNORD10	ENSG00000238917.1	0.023
291	chr13:42614175-42830714	DGKH	ENSG00000102780.12	0.023
292	chr11:94883702-94967268	SESN3	ENSG00000149212.6	0.023
293	chr2:235401684-235405697	ARL4C	ENSG00000188042.5	0.023
294	chr6:100956069-101329248	ASCC3	ENSG00000112249.9	0.023
295	chr11:104896169-104972158	CARD16	ENSG00000204397.3	0.023
296	chrX:151121595-151143152	GABRE	ENSG00000102287.12	0.023
297	chr20:19867164-19983101	RIN2	ENSG00000132669.8	0.023
298	chr10:70975088-71027904	RP11-227H15.4	ENSG00000229261.1	0.023
299	chrX:17755587-17773105	SCML1	ENSG00000047634.10	0.023
300	chr1:211916798-212027045	LPGAT1	ENSG00000123684.8	0.023
301	chr5:149546357-149564120	CDX1	ENSG00000113722.12	0.023
302	chr1:161475219-161648444	HSPA6	ENSG00000173110.6	0.023
303	chr7:127937737-127983962	RBM28	ENSG00000106344.4	0.023
304	chr13:95226307-95248511	TGDS	ENSG00000088451.6	0.023
305	chr10:135093134-135166187	ZNF511	ENSG00000198546.10	0.022
306	chr11:14665268-14892350	PDE3B	ENSG00000152270.4	0.022
307	chr3:142315228-142432506	PLS1	ENSG00000120756.8	0.022
308	chr7:95034174-95064510	PON2	ENSG00000105854.8	0.022
309	chr7:75931860-75933612	HSPB1	ENSG00000106211.8	0.022
310	chr3:149086808-149104370	TM4SF1	ENSG00000169908.6	0.022
311	chr12:6643092-6647537	RP5-940J5.9	ENSG00000269968.1	0.022
312	chr7:100951626-100954266	RP11-132A1.4	ENSG00000232445.1	0.022
313	chr2:62759717-62760194	AC092155.1	ENSG00000229503.1	0.022
314	chr10:112836789-112840658	ADRA2A	ENSG00000150594.5	0.022
315	chr16:66968346-67009051	CES3	ENSG00000172828.8	0.022
316	chrM:8365-9990	MT-CO3	ENSG00000198938.2	0.022
317	chr5:473350-524447	CTD-2228K2.7	ENSG00000225138.3	0.022
318	chr16:14860258-14863932	RP11-680G24.1	ENSG00000255037.1	0.022
319	chr13:72012097-72441330	H3F3BP1	ENSG00000236534.1	0.022
320	chr11:102391238-102401484	MMP7	ENSG00000137673.4	0.022
321	chr1:144146807-144224481	NBPF8	ENSG00000162825.12	0.022
322	chr1:20808883-20812713	CAMK2N1	ENSG00000162545.5	0.022
323	chr5:85913720-85916779	COX7C	ENSG00000127184.6	0.022

324	chr3:22423309-22424420	HMGB1P5	ENSG00000132967.9	0.022
325	chr22:24198613-24241117	MIF	ENSG00000240972.1	0.022
326	chr17:29224353-29286340	TEFM	ENSG00000172171.6	0.022
327	chr7:73007523-73038873	MLXIPL	ENSG00000009950.11	0.022
328	chr8:29952913-30041156	RPS15AP24	ENSG00000241511.1	0.022
329	chr7:87462882-87538856	DBF4	ENSG00000006634.3	0.022
330	chr5:27472398-27496508	LINC01021	ENSG00000250337.1	0.022
331	chr20:56884751-56942563	RAB22A	ENSG00000124209.3	0.022
332	chr16:54952774-54963101	CRNDE	ENSG00000245694.4	0.022
333	chr2:103236165-103327777	SLC9A2	ENSG00000115616.2	0.022
334	chr2:41370919-41384695	HNRNPA1P57	ENSG00000237442.3	0.022
335	chr20:47835883-47905797	ZFAS1	ENSG00000177410.8	0.022
336	chr16:89642175-89663654	CPNE7	ENSG00000178773.10	0.022
337	chr5:92953774-93447404	NPM1P27	ENSG00000249353.2	0.022
338	chrX:73164158-73513409	RP3-368A4.5	ENSG00000271430.1	0.022
339	chrX:118722299-118739858	NKRF	ENSG00000186416.8	0.022
340	chr19:12305829-12405702	ZNF44	ENSG00000197857.9	0.022
341	chr11:67351065-67354131	GSTP1	ENSG00000084207.11	0.022
342	chr16:2089815-2185899	PKD1	ENSG00000008710.13	0.022

Table S2 - The significantly enriched GO biological process (BP), molecular function (MF) and cellular component (CC) terms of the up regulated transcripts in CRC epithelial cells

GO category	GO term	FDR	P value
biological process	GO:0070972_protein localization to endoplasmic reticulum	6.94E-06	4.39E-10
biological process	GO:0006413_translational initiation	2.54E-05	3.55E-09
biological process	GO:0000184_nuclear-transcribed mRNA catabolic process, nonsense-mediated decay	2.54E-05	4.83E-09
biological process	GO:0006614_SRP-dependent cotranslational protein targeting to membrane	2.54E-05	6.44E-09
biological process	GO:0006613_cotranslational protein targeting to membrane	3.28E-05	1.04E-08
biological process	GO:0045047_protein targeting to ER	3.92E-05	1.49E-08
biological process	GO:0033365_protein localization to organelle	4.15E-05	1.84E-08
biological process	GO:0072599_establishment of protein localization to endoplasmic reticulum	4.16E-05	2.11E-08
biological process	GO:0090304_nucleic acid metabolic process	5.86E-05	3.34E-08
biological process	GO:0006402_mRNA catabolic process	5.94E-05	3.76E-08
biological process	GO:0000956_nuclear-transcribed mRNA catabolic process	0.000139	9.67E-08
biological process	GO:0006401_RNA catabolic process	0.00016	1.21E-07
biological process	GO:0031328_positive regulation of cellular biosynthetic process	0.000233	1.91E-07
biological process	GO:0022613_ribonucleoprotein complex biogenesis	0.000261	2.59E-07
biological process	GO:0006457_protein folding	0.000261	2.65E-07
biological process	GO:0009891_positive regulation of biosynthetic process	0.000261	2.76E-07
biological process	GO:0010629_negative regulation of gene expression	0.000261	2.82E-07
biological process	GO:0071157_negative regulation of cell cycle arrest	0.000261	2.98E-07
biological process	GO:0010605_negative regulation of macromolecule metabolic process	0.000435	5.42E-07
biological process	GO:0006979_response to oxidative stress	0.000435	5.51E-07
biological process	GO:0043069_negative regulation of programmed cell death	0.000435	5.78E-07
biological process	GO:0080135_regulation of cellular response to stress	0.000443	6.17E-07
biological process	GO:0072594_establishment of protein localization to organelle	0.000558	8.14E-07
biological process	GO:0016071_mRNA metabolic process	0.000558	8.56E-07
biological process	GO:0034645_cellular macromolecule biosynthetic process	0.000558	9.12E-07
biological process	GO:0009059_macromolecule biosynthetic process	0.000558	9.18E-07
biological process	GO:0006612_protein targeting to membrane	0.000587	1.00E-06
biological process	GO:0010557_positive regulation of macromolecule biosynthetic process	0.000761	1.38E-06
biological process	GO:0000302_response to reactive oxygen species	0.000761	1.40E-06
biological process	GO:0009892_negative regulation of metabolic process	0.000888	1.69E-06
biological process	GO:0043066_negative regulation of apoptotic process	0.000962	1.94E-06
biological process	GO:0019083_viral transcription	0.000962	1.99E-06
biological process	GO:0006725_cellular aromatic compound metabolic process	0.000962	2.01E-06
biological process	GO:0044249_cellular biosynthetic process	0.00102	2.19E-06
biological process	GO:0060548_negative regulation of cell death	0.001048	2.32E-06
biological process	GO:0016072_rRNA metabolic process	0.001152	2.66E-06
biological process	GO:0044419_interspecies interaction between organisms	0.001152	2.77E-06
biological process	GO:0009893_positive regulation of metabolic process	0.001152	2.77E-06
biological process	GO:0019439_aromatic compound catabolic process	0.001152	2.85E-06
biological process	GO:0010564_regulation of cell cycle process	0.001237	3.13E-06
biological process	GO:0006139_nucleobase-containing compound metabolic process	0.001323	3.44E-06
biological process	GO:2000738_positive regulation of stem cell differentiation	0.001323	3.52E-06
biological process	GO:0019080_viral gene expression	0.001365	3.73E-06
biological process	GO:0034655_nucleobase-containing compound catabolic process	0.001365	3.88E-06
biological process	GO:1901576_organic substance biosynthetic process	0.001365	3.97E-06
biological process	GO:0044265_cellular macromolecule catabolic process	0.001365	3.98E-06
biological process	GO:0051726_regulation of cell cycle	0.001518	4.52E-06
biological process	GO:1905323_telomerase holoenzyme complex assembly	0.001645	5.00E-06
biological process	GO:0044403_symbiosis, encompassing mutualism through parasitism	0.001905	5.91E-06
biological process	GO:0009058_biosynthetic process	0.001915	6.06E-06
biological process	GO:0033554_cellular response to stress	0.001971	6.36E-06
biological process	GO:0009057_macromolecule catabolic process	0.00209	6.88E-06
biological process	GO:0010604_positive regulation of macromolecule metabolic process	0.00216	7.38E-06
biological process	GO:1901360_organic cyclic compound metabolic process	0.00216	7.49E-06
biological process	GO:0042254_ribosome biogenesis	0.00216	7.62E-06
biological process	GO:0010468_regulation of gene expression	0.00216	7.66E-06
biological process	GO:0044085_cellular component biogenesis	0.002162	7.80E-06
biological process	GO:0016032_viral process	0.002342	8.73E-06
biological process	GO:0008219_cell death	0.002342	8.78E-06
biological process	GO:0034605_cellular response to heat	0.002342	8.90E-06
biological process	GO:0046483_heterocycle metabolic process	0.002431	9.39E-06
biological process	GO:0046700_heterocycle catabolic process	0.002566	1.01E-05
biological process	GO:1900034_regulation of cellular response to heat	0.002566	1.05E-05
biological process	GO:0044270_cellular nitrogen compound catabolic process	0.002566	1.05E-05
biological process	GO:0090150_establishment of protein localization to membrane	0.002566	1.06E-05
biological process	GO:0043588_skin development	0.002978	1.24E-05
biological process	GO:0034599_cellular response to oxidative stress	0.003194	1.35E-05
biological process	GO:0060255_regulation of macromolecule metabolic process	0.003247	1.41E-05
biological process	GO:0016070_RNA metabolic process	0.003247	1.42E-05
biological process	GO:0051179_localization	0.00333	1.48E-05
biological process	GO:0071840_cellular component organization or biogenesis	0.003483	1.57E-05
biological process	GO:0031325_positive regulation of cellular metabolic process	0.003919	1.79E-05
biological process	GO:0048519_negative regulation of biological process	0.004065	1.88E-05

biological process	GO:0048608_reproductive structure development	0.004065	1.90E-05
biological process	GO:0009889_regulation of biosynthetic process	0.004185	1.99E-05
biological process	GO:0010467_gene expression	0.004185	2.02E-05
biological process	GO:0061458_reproductive system development	0.004185	2.04E-05
biological process	GO:0012501_programmed cell death	0.004208	2.08E-05
biological process	GO:0019222_regulation of metabolic process	0.004211	2.11E-05
biological process	GO:0042542_response to hydrogen peroxide	0.004765	2.41E-05
biological process	GO:1901361_organic cyclic compound catabolic process	0.00489	2.53E-05
biological process	GO:0010628_positive regulation of gene expression	0.00489	2.54E-05
biological process	GO:0045787_positive regulation of cell cycle	0.005203	2.73E-05
biological process	GO:0007050_cell cycle arrest	0.006229	3.32E-05
biological process	GO:0045935_positive regulation of nucleobase-containing compound metabolic process	0.006229	3.35E-05
biological process	GO:0060249_anatomical structure homeostasis	0.006544	3.56E-05
biological process	GO:0045934_negative regulation of nucleobase-containing compound metabolic process	0.006777	3.73E-05
biological process	GO:0072163_mesonephric epithelium development	0.006784	3.87E-05
biological process	GO:0072164_mesonephric tubule development	0.006784	3.87E-05
biological process	GO:0044271_cellular nitrogen compound biosynthetic process	0.006784	3.89E-05
biological process	GO:0034613_cellular protein localization	0.006784	3.92E-05
biological process	GO:0051641_cellular localization	0.006784	3.97E-05
biological process	GO:0006364_rRNA processing	0.006784	3.99E-05
biological process	GO:0070727_cellular macromolecule localization	0.007219	4.31E-05
biological process	GO:0014032_neural crest cell development	0.007219	4.34E-05
biological process	GO:0048754_branching morphogenesis of an epithelial tube	0.007513	4.57E-05
biological process	GO:0042592_homeostatic process	0.007684	4.72E-05
biological process	GO:0006915_apoptotic process	0.007706	4.78E-05
biological process	GO:0001823_mesonephros development	0.007735	4.85E-05
biological process	GO:2000573_positive regulation of DNA biosynthetic process	0.008648	5.48E-05
biological process	GO:0006259_DNA metabolic process	0.008648	5.53E-05
biological process	GO:0014031_mesenchymal cell development	0.009059	5.91E-05
biological process	GO:0048864_stem cell development	0.009059	5.91E-05
biological process	GO:0007004_telomere maintenance via telomerase	0.010299	6.84E-05
biological process	GO:0031326_regulation of cellular biosynthetic process	0.010299	6.85E-05
biological process	GO:0034641_cellular nitrogen compound metabolic process	0.010299	6.91E-05
biological process	GO:2000826_regulation of heart morphogenesis	0.010453	7.08E-05
biological process	GO:0007049_cell cycle	0.010926	7.47E-05
biological process	GO:0009408_response to heat	0.011279	7.78E-05
biological process	GO:0014033_neural crest cell differentiation	0.01131	7.88E-05
biological process	GO:0071156_regulation of cell cycle arrest	0.01223	8.59E-05
biological process	GO:0007346_regulation of mitotic cell cycle	0.012284	8.71E-05
biological process	GO:0043170_macromolecule metabolic process	0.013141	9.48E-05
biological process	GO:0044248_cellular catabolic process	0.013141	9.48E-05
biological process	GO:0071496_cellular response to external stimulus	0.01318	9.60E-05
biological process	GO:0030155_regulation of cell adhesion	0.013668	0.000102
biological process	GO:0010558_negative regulation of macromolecule biosynthetic process	0.013668	0.000102
biological process	GO:0010556_regulation of macromolecule biosynthetic process	0.013668	0.000102
biological process	GO:0080134_regulation of response to stress	0.014357	0.000109
biological process	GO:0003197_endocardial cushion development	0.014357	0.000109
biological process	GO:0006278_RNA-dependent DNA biosynthetic process	0.01437	0.00011
biological process	GO:0072190_ureter urothelium development	0.014862	0.000121
biological process	GO:0072197_ureter morphogenesis	0.014862	0.000121
biological process	GO:0072717_cellular response to actinomycin D	0.014862	0.000121
biological process	GO:1905292_regulation of neural crest cell differentiation	0.014862	0.000121
biological process	GO:1905294_positive regulation of neural crest cell differentiation	0.014862	0.000121
biological process	GO:1905310_regulation of cardiac neural crest cell migration involved in outflow tract morphogenesis	0.014862	0.000121
biological process	GO:1905312_positive regulation of cardiac neural crest cell migration involved in outflow tract morphogenesis	0.014862	0.000121
biological process	GO:0033036_macromolecule localization	0.014862	0.000121
biological process	GO:2000112_regulation of cellular macromolecule biosynthetic process	0.014975	0.000123
biological process	GO:0072657_protein localization to membrane	0.015138	0.000126
biological process	GO:0006986_response to unfolded protein	0.015138	0.000127
biological process	GO:0072028_nephron morphogenesis	0.01544	0.000133
biological process	GO:0045723_positive regulation of fatty acid biosynthetic process	0.01544	0.000134
biological process	GO:0071236_cellular response to antibiotic	0.01544	0.000134
biological process	GO:0045892_negative regulation of transcription, DNA-templated	0.01544	0.000134
biological process	GO:0065003_macromolecular complex assembly	0.01544	0.000134
biological process	GO:2000113_negative regulation of cellular macromolecule biosynthetic process	0.016065	0.000141
biological process	GO:0010833_telomere maintenance via telomere lengthening	0.016065	0.000141
biological process	GO:0019219_regulation of nucleobase-containing compound metabolic process	0.016089	0.000143
biological process	GO:0044260_cellular macromolecule metabolic process	0.016716	0.000149
biological process	GO:0061138_morphogenesis of a branching epithelium	0.017363	0.000156
biological process	GO:0008544_epidermis development	0.017431	0.000158
biological process	GO:0045786_negative regulation of cell cycle	0.018676	0.00017
biological process	GO:1901031_regulation of response to reactive oxygen species	0.019135	0.000176
biological process	GO:0007167_enzyme linked receptor protein signaling pathway	0.020843	0.000193
biological process	GO:0098609_cell-cell adhesion	0.021405	0.0002

biological process	GO:0042475_odontogenesis of dentin-containing tooth	0.021405	0.000201
biological process	GO:0051052_regulation of DNA metabolic process	0.021472	0.000203
biological process	GO:0008152_metabolic process	0.021775	0.000207
biological process	GO:0072132_mesenchyme morphogenesis	0.021872	0.000209
biological process	GO:2000145_regulation of cell motility	0.022547	0.000217
biological process	GO:0065008_regulation of biological quality	0.02263	0.00022
biological process	GO:1903507_negative regulation of nucleic acid-templated transcription	0.02263	0.000221
biological process	GO:0006605_protein targeting	0.022816	0.000224
biological process	GO:1902679_negative regulation of RNA biosynthetic process	0.022816	0.000225
biological process	GO:0071363_cellular response to growth factor stimulus	0.022832	0.000228
biological process	GO:0043933_macromolecular complex subunit organization	0.022832	0.000234
biological process	GO:0010941_regulation of cell death	0.022832	0.000236
biological process	GO:0001763_morphogenesis of a branching structure	0.022832	0.00024
biological process	GO:0060686_negative regulation of prostatic bud formation	0.022832	0.00024
biological process	GO:0072125_negative regulation of glomerular mesangial cell proliferation	0.022832	0.00024
biological process	GO:0072191_ureter smooth muscle development	0.022832	0.00024
biological process	GO:0072193_ureter smooth muscle cell differentiation	0.022832	0.00024
biological process	GO:0072716_response to actinomycin D	0.022832	0.00024
biological process	GO:0090194_negative regulation of glomerulus development	0.022832	0.00024
biological process	GO:0051234_establishment of localization	0.022832	0.000241
biological process	GO:0030850_prostate gland development	0.022973	0.000247
biological process	GO:0046677_response to antibiotic	0.022973	0.000247
biological process	GO:0035966_response to topologically incorrect protein	0.022973	0.000247
biological process	GO:0009890_negative regulation of biosynthetic process	0.023494	0.000254
biological process	GO:0072006_nephron development	0.023546	0.000257
biological process	GO:0006518_peptide metabolic process	0.023546	0.000258
biological process	GO:0051173_positive regulation of nitrogen compound metabolic process	0.023897	0.000263
biological process	GO:0003006_developmental process involved in reproduction	0.023932	0.000265
biological process	GO:0072073_kidney epithelium development	0.023976	0.000267
biological process	GO:0022402_cell cycle process	0.02431	0.000272
biological process	GO:0009056_catabolic process	0.024652	0.000278
biological process	GO:0051704_multi-organism process	0.024721	0.000282
biological process	GO:0006950_response to stress	0.024721	0.000282
biological process	GO:0034660_ncRNA metabolic process	0.024721	0.000283
biological process	GO:0035272_exocrine system development	0.025113	0.000289
biological process	GO:0070301_cellular response to hydrogen peroxide	0.02517	0.000292
biological process	GO:0031396_regulation of protein ubiquitination	0.025719	0.0003
biological process	GO:0060993_kidney morphogenesis	0.026056	0.000307
biological process	GO:1902882_regulation of response to oxidative stress	0.026056	0.000307
biological process	GO:0006412_translation	0.027549	0.000327
biological process	GO:0065009_regulation of molecular function	0.027549	0.000328
biological process	GO:0070848_response to growth factor	0.028377	0.00034
biological process	GO:0044267_cellular protein metabolic process	0.028973	0.000348
biological process	GO:0030334_regulation of cell migration	0.029769	0.00036
biological process	GO:0060688_regulation of morphogenesis of a branching structure	0.029826	0.000363
biological process	GO:0071824_protein-DNA complex subunit organization	0.029921	0.000366
biological process	GO:0048732_gland development	0.030271	0.000372
biological process	GO:0008104_protein localization	0.030316	0.000374
biological process	GO:0071897_DNA biosynthetic process	0.030396	0.000377
biological process	GO:0001657_ureteric bud development	0.031435	0.000392
biological process	GO:0097190_apoptotic signaling pathway	0.032176	0.000405
biological process	GO:0010035_response to inorganic substance	0.032176	0.000405
biological process	GO:0044092_negative regulation of molecular function	0.032282	0.000409
biological process	GO:2001234_negative regulation of apoptotic signaling pathway	0.032304	0.000411
biological process	GO:0022610_biological adhesion	0.032363	0.000416
biological process	GO:0010332_response to gamma radiation	0.032363	0.000418
biological process	GO:0048522_positive regulation of cellular process	0.032363	0.000419
biological process	GO:0008283_cell proliferation	0.032363	0.00042
biological process	GO:0043043_peptide biosynthetic process	0.03487	0.000455
biological process	GO:0031668_cellular response to extracellular stimulus	0.035104	0.00046
biological process	GO:0050821_protein stabilization	0.03511	0.000462
biological process	GO:0042026_protein refolding	0.035146	0.000465
biological process	GO:0050790_regulation of catalytic activity	0.035146	0.000467
biological process	GO:0045859_regulation of protein kinase activity	0.03524	0.000472
biological process	GO:0051054_positive regulation of DNA metabolic process	0.03524	0.000473
biological process	GO:0000723_telomere maintenance	0.035484	0.000478
biological process	GO:0007169_transmembrane receptor protein tyrosine kinase signaling pathway	0.035502	0.000481
biological process	GO:2000278_regulation of DNA biosynthetic process	0.036299	0.000494
biological process	GO:0040012_regulation of locomotion	0.036562	0.0005
biological process	GO:0051253_negative regulation of RNA metabolic process	0.03657	0.000502
biological process	GO:0034614_cellular response to reactive oxygen species	0.037106	0.000512
biological process	GO:0019538_protein metabolic process	0.037387	0.000518
biological process	GO:0031327_negative regulation of cellular biosynthetic process	0.039241	0.000551
biological process	GO:0009636_response to toxic substance	0.039241	0.000557

biological process	GO:0006260_DNA replication	0.039241	0.000558
biological process	GO:1903320_regulation of protein modification by small protein conjugation or removal	0.039241	0.000558
biological process	GO:0009968_negative regulation of signal transduction	0.039241	0.000558
biological process	GO:0008284_positive regulation of cell proliferation	0.039241	0.000562
biological process	GO:0010648_negative regulation of cell communication	0.039241	0.000563
biological process	GO:0051172_negative regulation of nitrogen compound metabolic process	0.039241	0.000567
biological process	GO:0051270_regulation of cellular component movement	0.039241	0.000568
biological process	GO:0097193_intrinsic apoptotic signaling pathway	0.039241	0.000571
biological process	GO:0043067_regulation of programmed cell death	0.039241	0.000578
biological process	GO:0023057_negative regulation of signaling	0.039241	0.000584
biological process	GO:0009266_response to temperature stimulus	0.039241	0.000587
biological process	GO:0060740_prostate gland epithelium morphogenesis	0.039241	0.000592
biological process	GO:0072273_metanephric nephron morphogenesis	0.039241	0.000592
biological process	GO:0060685_regulation of prostatic bud formation	0.039241	0.000596
biological process	GO:0061046_regulation of branching involved in lung morphogenesis	0.039241	0.000596
biological process	GO:1901723_negative regulation of cell proliferation involved in kidney development	0.039241	0.000596
biological process	GO:1902338_negative regulation of apoptotic process involved in morphogenesis	0.039241	0.000596
biological process	GO:1904746_negative regulation of apoptotic process involved in development	0.039241	0.000596
biological process	GO:2000138_positive regulation of cell proliferation involved in heart morphogenesis	0.039241	0.000596
biological process	GO:0045893_positive regulation of transcription, DNA-templated	0.040152	0.000615
biological process	GO:1903508_positive regulation of nucleic acid-templated transcription	0.040152	0.000615
biological process	GO:1902680_positive regulation of RNA biosynthetic process	0.040271	0.000621
biological process	GO:0000278_mitotic cell cycle	0.040271	0.000622
biological process	GO:0034504_protein localization to nucleus	0.040896	0.000634
biological process	GO:0051338_regulation of transferase activity	0.042143	0.000656
biological process	GO:0070198_protein localization to chromosome, telomeric region	0.042235	0.000663
biological process	GO:0071480_cellular response to gamma radiation	0.042235	0.000663
biological process	GO:0030330_DNA damage response, signal transduction by p53 class mediator	0.042268	0.000669
biological process	GO:0072009_nephron epithelium development	0.042268	0.000669
biological process	GO:0031400_negative regulation of protein modification process	0.042356	0.000673
biological process	GO:0031324_negative regulation of cellular metabolic process	0.044457	0.000709
biological process	GO:0042303_molting cycle	0.045181	0.000727
biological process	GO:0042633_hair cycle	0.045181	0.000727
biological process	GO:0032200_telomere organization	0.045295	0.000731
biological process	GO:0060512_prostate gland morphogenesis	0.045435	0.000739
biological process	GO:0071634_regulation of transforming growth factor beta production	0.045435	0.000739
biological process	GO:1901701_cellular response to oxygen-containing compound	0.046	0.000753
biological process	GO:0007219_Notch signaling pathway	0.046	0.000754
biological process	GO:0061077_chaperone-mediated protein folding	0.04802	0.00079
biological process	GO:0003203_endocardial cushion morphogenesis	0.049361	0.000821
biological process	GO:0071604_transforming growth factor beta production	0.049361	0.000821
biological process	GO:0003253_cardiac neural crest cell migration involved in outflow tract morphogenesis	0.049361	0.000831
biological process	GO:0032808_lacrimal gland development	0.049361	0.000831
biological process	GO:0060687_regulation of branching involved in prostate gland morphogenesis	0.049361	0.000831
biological process	GO:0060710_chorio-allantoic fusion	0.049361	0.000831
biological process	GO:0065007_biological regulation	0.049665	0.00084
biological process	GO:0018193_peptidyl-amino acid modification	0.049665	0.000843
molecular function	GO:0003723_RNA binding	0.000887	1.93E-07
molecular function	GO:0003735_structural constituent of ribosome	0.001626	8.91E-07
molecular function	GO:1901363_heterocyclic compound binding	0.001626	1.06E-06
molecular function	GO:0097159_organic cyclic compound binding	0.002099	1.82E-06
molecular function	GO:0003676_nucleic acid binding	0.006516	8.09E-06
molecular function	GO:0051082_unfolded protein binding	0.006516	8.49E-06
molecular function	GO:0019843_rRNA binding	0.025532	3.88E-05
cellular component	GO:0022626_cytosolic ribosome	1.44E-07	7.59E-11
cellular component	GO:0044391_ribosomal subunit	1.38E-05	1.47E-08
cellular component	GO:0044445_cytosolic part	1.38E-05	2.18E-08
cellular component	GO:0031974_membrane-enclosed lumen	1.98E-05	6.24E-08
cellular component	GO:0043233_organelle lumen	1.98E-05	6.24E-08
cellular component	GO:0070013_intracellular organelle lumen	1.98E-05	6.24E-08
cellular component	GO:0044428_nuclear part	2.81E-05	1.03E-07
cellular component	GO:0005840_ribosome	4.03E-05	1.70E-07
cellular component	GO:0031981_nuclear lumen	8.91E-05	4.22E-07
cellular component	GO:0070062_extracellular exosome	8.92E-05	4.69E-07
cellular component	GO:1903561_extracellular vesicle	9.00E-05	5.58E-07
cellular component	GO:0043230_extracellular organelle	9.00E-05	5.68E-07
cellular component	GO:0060205_cytoplasmic vesicle lumen	0.000124	8.84E-07
cellular component	GO:0031983_vesicle lumen	0.000124	9.12E-07
cellular component	GO:0030529_intracellular ribonucleoprotein complex	0.000185	1.48E-06
cellular component	GO:1990904_ribonucleoprotein complex	0.000185	1.56E-06
cellular component	GO:0022625_cytosolic large ribosomal subunit	0.000193	1.72E-06
cellular component	GO:0005634_nucleus	0.000218	2.06E-06
cellular component	GO:0071682_endocytic vesicle lumen	0.000432	4.43E-06
cellular component	GO:0005654_nucleoplasm	0.000432	4.54E-06

cellular component	GO:0022627_cytosolic small ribosomal subunit	0.000647	7.15E-06
cellular component	GO:1904813_ficolin-1-rich granule lumen	0.000842	9.74E-06
cellular component	GO:0005615_extracellular space	0.002121	2.56E-05
cellular component	GO:0031982_vesicle	0.002828	3.57E-05
cellular component	GO:0032991_macromolecular complex	0.004883	6.42E-05
cellular component	GO:0015934_large ribosomal subunit	0.004914	6.72E-05
cellular component	GO:0015935_small ribosomal subunit	0.004914	7.20E-05
cellular component	GO:0070161_anchoring junction	0.004914	7.23E-05
cellular component	GO:0044446_intracellular organelle part	0.005934	9.51E-05
cellular component	GO:0044421_extracellular region part	0.005934	9.55E-05
cellular component	GO:0005829_cytosol	0.005934	9.67E-05
cellular component	GO:0101002_ficolin-1-rich granule	0.007481	0.000126
cellular component	GO:0005925_focal adhesion	0.007488	0.00013
cellular component	GO:0034774_secretory granule lumen	0.00751	0.000136
cellular component	GO:0005924_cell-substrate adherens junction	0.00751	0.000138
cellular component	GO:0030055_cell-substrate junction	0.008084	0.000153
cellular component	GO:0031838_haptoglobin-hemoglobin complex	0.010905	0.000212
cellular component	GO:0005912_adherens junction	0.011528	0.00023
cellular component	GO:0044422_organelle part	0.012062	0.000247
cellular component	GO:0005576_extracellular region	0.014688	0.000309
cellular component	GO:0043227_membrane-bounded organelle	0.029999	0.000647
cellular component	GO:0044433_cytoplasmic vesicle part	0.039654	0.000876
cellular component	GO:0031410_cytoplasmic vesicle	0.049798	0.00113
cellular component	GO:0097708_intracellular vesicle	0.049798	0.001152

Table S3 - The significantly enriched GO biological process (BP), molecular function (MF) and cellular component (CC) terms of the down regulated transcripts in CRC epithelial cells

GO category	GO term	FDR	P value
biological process	GO:0071294_cellular response to zinc ion	5.83E-05	3.69E-09
biological process	GO:0010043_response to zinc ion	0.000962	1.61E-07
biological process	GO:0006811_ion transport	0.000962	1.84E-07
biological process	GO:0015672_monovalent inorganic cation transport	0.000962	2.44E-07
biological process	GO:0015701_bicarbonate transport	0.003746	1.19E-06
biological process	GO:1902600_hydrogen ion transmembrane transport	0.004886	1.91E-06
biological process	GO:0006821_chloride transport	0.004886	2.36E-06
biological process	GO:0055067_monovalent inorganic cation homeostasis	0.004886	2.47E-06
biological process	GO:0071248_cellular response to metal ion	0.005363	3.44E-06
biological process	GO:0015992_proton transport	0.005363	3.63E-06
biological process	GO:0006818_hydrogen transport	0.005363	4.04E-06
biological process	GO:0071276_cellular response to cadmium ion	0.005363	4.07E-06
biological process	GO:0098660_inorganic ion transmembrane transport	0.007924	6.52E-06
biological process	GO:0007586_digestion	0.008341	7.39E-06
biological process	GO:0015698_inorganic anion transport	0.010338	9.82E-06
biological process	GO:0071241_cellular response to inorganic substance	0.011636	1.18E-05
biological process	GO:0006810_transport	0.022374	2.41E-05
biological process	GO:0030004_cellular monovalent inorganic cation homeostasis	0.023038	2.63E-05
biological process	GO:0034220_ion transmembrane transport	0.032273	3.88E-05
biological process	GO:0055085_transmembrane transport	0.034959	4.51E-05
biological process	GO:0051234_establishment of localization	0.034959	4.65E-05
biological process	GO:0006812_cation transport	0.039054	5.61E-05
biological process	GO:0010038_response to metal ion	0.039054	5.69E-05
molecular function	GO:0015078_hydrogen ion transmembrane transporter activity	0.007706	1.67E-06
molecular function	GO:0022857_transmembrane transporter activity	0.012589	5.47E-06
molecular function	GO:0005215_transporter activity	0.01586	1.14E-05
molecular function	GO:0022891_substrate-specific transmembrane transporter activity	0.01586	1.59E-05
molecular function	GO:0015075_ion transmembrane transporter activity	0.01586	1.84E-05
molecular function	GO:0008270_zinc ion binding	0.01586	2.11E-05
molecular function	GO:0022892_substrate-specific transporter activity	0.01586	2.76E-05
molecular function	GO:0015077_monovalent inorganic cation transmembrane transporter activity	0.01586	3.56E-05
molecular function	GO:0004022_alcohol dehydrogenase (NAD) activity	0.01586	3.91E-05
molecular function	GO:0004129_cytochrome-c oxidase activity	0.01586	4.13E-05
molecular function	GO:0015002_heme-copper terminal oxidase activity	0.01586	4.13E-05
molecular function	GO:0016676_oxidoreductase activity, acting on a heme group of donors, oxygen as acceptor	0.01586	4.13E-05
molecular function	GO:0005254_chloride channel activity	0.015968	4.64E-05
molecular function	GO:0016675_oxidoreductase activity, acting on a heme group of donors	0.015968	4.86E-05
molecular function	GO:0015108_chloride transmembrane transporter activity	0.030417	9.99E-05
molecular function	GO:0015103_inorganic anion transmembrane transporter activity	0.030417	0.000107
molecular function	GO:0005253_anion channel activity	0.030417	0.000112
molecular function	GO:0004089_carbonate dehydratase activity	0.042047	0.000164
cellular component	GO:0070062_extracellular exosome	9.08E-06	1.11E-08
cellular component	GO:1903561_extracellular vesicle	9.08E-06	1.40E-08
cellular component	GO:0043230_extracellular organelle	9.08E-06	1.43E-08
cellular component	GO:0005615_extracellular space	3.08E-05	6.52E-08
cellular component	GO:0044421_extracellular region part	3.08E-05	8.11E-08
cellular component	GO:0005576_extracellular region	0.000321	1.01E-06
cellular component	GO:0031982_vesicle	0.00176	6.48E-06
cellular component	GO:0005743_mitochondrial inner membrane	0.013524	5.69E-05
cellular component	GO:0042589_zymogen granule membrane	0.019482	9.22E-05
cellular component	GO:0071944_cell periphery	0.020388	0.000107
cellular component	GO:0019866_organelle inner membrane	0.03164	0.000189
cellular component	GO:0042588_zymogen granule	0.03164	0.0002
cellular component	GO:0030667_secretory granule membrane	0.035332	0.000241

Table S4 - The 55 CRC signature genes from Chu et al.

Index	Gene Name
1	CA7
2	SPIB
3	GUCA2B
4	AQP8
5	IL6R
6	SPP1
7	TCN1
8	CWH43
9	SST
10	KIAA1199
11	SLC4A4
12	CHP2
13	GCG
14	NR3C2
15	NFE2L3
16	CLDN1
17	C9orf125
18	CPM
19	CLDN8
20	MMP7
21	EDN3
22	GUCA2A
23	CDH3
24	FAM55D
25	NR3C1
26	PYY
27	SLC30A10
28	BEST2
29	CLCA1
30	TRPM6
31	SCNN1B
32	THRB
33	ABCG2
34	SPINK5
35	GALNT6
36	CHST5
37	NUP153
38	DEFA6
39	SLC7A5
40	ZG16
41	C6orf105
42	HP

43	MUC2
44	KLK11
45	AHCYL2
46	CA1
47	H3F3A
48	CLCA4
49	BTNL3
50	FCGBP
51	MS4A12
52	CA4
53	CD177
54	SLC26A3
55	MT1M
